# The Influence of Chronic Liver Diseases on Hepatic Vasculature: A Liver-on-a-chip Review

**DOI:** 10.3390/mi11050487

**Published:** 2020-05-09

**Authors:** Alican Özkan, Danielle Stolley, Erik N. K. Cressman, Matthew McMillin, Sharon DeMorrow, Thomas E. Yankeelov, Marissa Nichole Rylander

**Affiliations:** 1 Department of Mechanical Engineering, The University of Texas, Austin, TX 78712, USA; mnr@austin.utexas.edu; 2 Department of Biomedical Engineering, The University of Texas, Austin, TX 78712, USA; danielle.stolley@austin.utexas.edu (D.S.); thomas.yankeelov@utexas.edu (T.E.Y.); 3 Department of Interventional Radiology, The University of Texas MD Anderson Cancer Center, Houston, TX 77030, USA; ECressman@mdanderson.org; 4 Department of Internal Medicine, Dell Medical School, The University of Texas at Austin, Austin, TX 78713, USA; matthew.mcmillin@austin.utexas.edu (M.M.); sharon.demorrow@austin.utexas.edu (S.D.); 5 Central Texas Veterans Health Care System, Temple, TX 76504, USA; 6 Division of Pharmacology and Toxicology, College of Pharmacy, The University of Texas at Austin, Austin, TX 78712, USA; 7 Oden Institute for Computational Engineering and Sciences, The University of Texas, Austin, TX 78712, USA; 8 Departments of Diagnostic Medicine, The University of Texas, Austin, TX 78712, USA; 9 Department of Oncology, The University of Texas, Austin, TX 78712, USA; 10 Livestrong Cancer Institutes, Dell Medical School, The University of Texas, Austin, TX 78712, USA

**Keywords:** microfluidics, organ-on-a-chip, tissue engineering, hepatology, vascular diseases, chronic liver diseases

## Abstract

In chronic liver diseases and hepatocellular carcinoma, the cells and extracellular matrix of the liver undergo significant alteration in response to chronic injury. Recent literature has highlighted the critical, but less studied, role of the liver vasculature in the progression of chronic liver diseases. Recent advancements in liver-on-a-chip systems has allowed in depth investigation of the role that the hepatic vasculature plays both in response to, and progression of, chronic liver disease. In this review, we first introduce the structure, gradients, mechanical properties, and cellular composition of the liver and describe how these factors influence the vasculature. We summarize state-of-the-art vascularized liver-on-a-chip platforms for investigating biological models of chronic liver disease and their influence on the liver sinusoidal endothelial cells of the hepatic vasculature. We conclude with a discussion of how future developments in the field may affect the study of chronic liver diseases, and drug development and testing.

## 1. Introduction

The liver is the largest solid internal organ in the human body and plays an important role in sustaining regular physiological activities. It has a significant role in metabolism of nutrients and detoxification of exogenous compounds [1,2]. Because of this, the liver is a common site for both acute and chronic injury [3]. While acute injuries of the liver may resolve, prolonged exposure to exogenous compounds (alcohol, high-fat nutrients, or chemotherapeutics) or viral hepatitis can lead to the development of chronic liver diseases. Comprising over half of the cellular population of the liver, hepatocytes are commonly the focus of studies into the pathogenesis of chronic liver diseases. Hepatocytes perform the majority of the liver’s functions, including metabolism, detoxification, and protein and bile synthesis. They also serve as the host population for viral hepatitis that can induce significant cellular dysregulation [4]. While the importance of hepatocytes in liver function and disease progression cannot be overstated, non-parenchymal cells such as those that comprise the hepatic vasculature also play a significant role in the healthy and diseased liver microenvironment.

Efforts to study the role of the vascular component in response to chronic liver disease have many challenges. Due to the multiple cell types contributing to this complex microenvironment, which is composed of multiple regions, gradients, and hemodynamics, in vivo animal models have been at the forefront of liver disease research and have been utilized for pharmaceutical testing and cellular investigations. The primary limitations of these models, however, are the inability to perform as detailed mechanistic studies due to the lack of tunability and to closely investigate the dynamics of disease progression [5,6]. For that reason, there has been increasing interest around engineering the hepatic structure using in vitro liver-on-a-chip systems. These liver-on-a-chip systems have shown great promise in recapitulating the hepatic response in a representative system that would permit high resolution and dynamic investigations in tunable systems.

With recent advances in microfluidics, the inclusion of vascular components in engineered in vitro systems allows the investigation of hemodynamics and the permeability of drugs and solutes through the vasculature to hepatocytes. This incorporation of hepatic vasculature can also benefit hepatocytes by helping them retain their functionality relative to that found in the in vivo setting. Recent literature has demonstrated that the inclusion of a vascular component of liver sinusoid endothelial cells (LSECs), with or without continuous flow and physiological wall shear stress, helps to sustain the correct morphology of hepatocytes in vitro [7]. Incorporation of the endothelium has several challenges since it requires introduction of acting wall shear stress via continuous flow provided by a pump or pressure gradient to maintain their elongated native morphology and functionality, which is cost prohibitive due to excessive consumption of culture media [8,9]. In addition, incorporation of flow promotes upregulation of functional protein secretion such as albumin, urea, and numerous drug metabolism enzymes of hepatocytes, which matches better with the real physiological conditions [10,11]. It is known that during chronic liver injury, the vascular component and incorporated LSECs may lose their functionality and their selectivity, which can accelerate liver damage [12]. For that reason, it is crucial to investigate the exact role that the hepatic vasculature plays both in response to, and during the progression of, chronic liver disease.

Traditionally, liver models used for in vitro studies have included 2D monolayers and static 3D methods, such as organoids, and spheroids [8,13]. Dynamic 3D models allow the incorporation of microfluidics, thus allowing the expansion of study into vascularized liver-on-a-chip systems. This approach enables the investigation of endothelium during different disease states under physiological flow conditions. To date, previous reviews have discussed the differences in fabrication methods, high throughput parallelization [14,15], and hepatic drug metabolism and response [16,17,18]. However, there is no comprehensive review of the impact of liver diseases on the vascular component, or the vascular component’s contribution to disease progression.

In this review, we first introduce the liver microenvironment, including cellular composition, microenvironmental properties, specific cellular roles, and current available cell sources for experimentation. We highlight the oxygen and nutrient gradients inside the liver and how they impact the hepatic vasculature. We then briefly discuss how the stiffness of the liver changes in response to chronic liver diseases. We introduce vascularized liver-on-a-chips categorized under different chronic liver diseases or presentations including fatty liver disease, viral hepatitis, fibrosis and cirrhosis, and hepatocellular carcinoma (HCC). We further discuss how these pathologies have been implemented in vitro, how they impact the hepatic vasculature, and the role LSECs play in disease progression. Finally, we summarize the current applications and the challenges facing future investigations of hepatic vasculature study.

## 2. Structure and Function of the Liver

The liver’s blood supply stems from the hepatic artery and portal vein [19,20]. Under most conditions, the hepatic artery approximately supplies 25% of blood through arterioles, and the portal vein supplies 75%. The portal vein, hepatic artery, and bile ducts (responsible for lymphatic drainage of the liver and surrounded by cholangiocytes), form the portal triad. Arrays of these triads form discrete polygonal liver lobules when examined microscopically. Within these liver lobules, the portal vein and hepatic artery combine near the periphery, contribution their flow to the channels of the liver called the liver sinusoids with flow directed to a central draining vein. These sinusoids have an approximate length of 250 μm and a variable diameter of 7–15 μm [19]. This vascular structure can regulate the blood flow to change nutrient delivery between the circulatory system and surrounding hepatocytes [21]. The bile duct, which is surrounded by cholangiocytes flows in the opposite direction of the liver sinusoid that carries bile from the liver to the gallbladder [22]. 

The extracellular matrix (ECM) of the liver provides mechanical integrity and is critical for cell proliferation, migration, differentiation, and protein expression. As such, changes in the ECM structure and stiffness, usually in response to chronic liver disease and fibrosis, have been shown to regulate vascular structure, integrity and functionality [9,23]. The ECM provides structure to all organs; however, a very limited amount is present within the healthy human liver [24]. The most commonly found ECM proteins are collagens including types I, III, and V [25,26].

**Figure 1:**
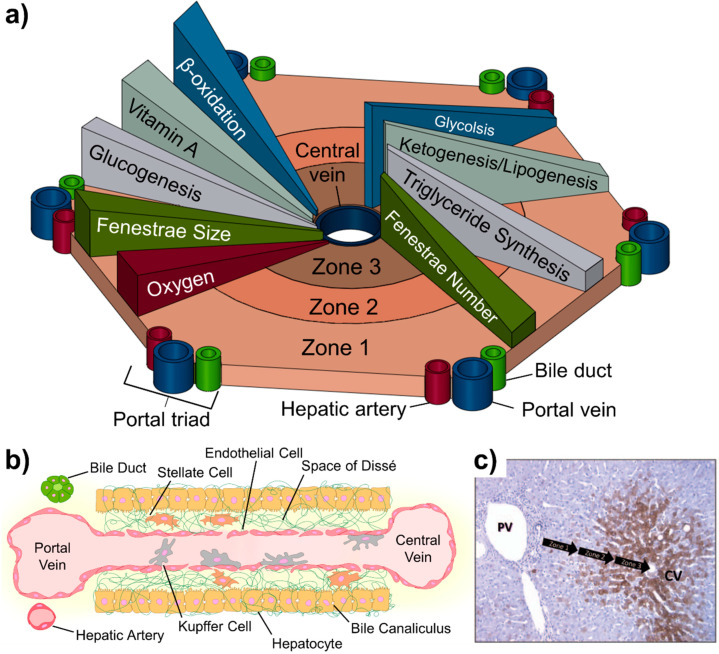
The liver lobule and the associated anatomy. (**a**) Biochemical pathways, gradients and endothelial properties alternation across the zones of liver lobule. Zone 1 is defined as the region closest to the “portal triad,” consisting of the portal vein, the hepatic artery, and the bile duct. The innermost zone is located near the central vein and is referred to as the pericentral region. Different anabolic and catabolic pathways are differentially active in different zones. A key “zonation modulator,” the Wnt/β-catenin and triglycerides (TG) pathways are active in the pericentral region near the central vein. The glucagon pathway, in contrast, displays its highest activity near the periportal region. (**b**) Composition of the liver sinusoid. (**c**) Variation of CYP3A4 expression across the lobule. Metabolic activity (brown) increases from the portal vein to the central vein. Reproduced from [35] with permission from Taylor & Francis Group.

The liver lobule is composed of three main zones (1, 2 and 3) that are concentrically segmented based on their proximity to the central vein and nearest portal triads that provide inflow (Figure 1a,b). These zones include the LSEC, hepatocytes, and other non-parenchymal cells that are found along the path of liver sinusoid starting at the portal triad and ending at the central vein [27]. Zone 1 is the periportal region that starts with the portal triad. Zone 2 is the mid lobular region that, compared to other zones, has somewhat more a balanced protein and nutrient gradient. Lastly, Zone 3 is the pericentral region that consists of the area surround the central draining vein. 

The heterogeneous configuration of cells along the liver sinusoid presents various chemical and nutrient gradients. The effect of this gradually changes in functional expression and metabolic activity of liver-specific cells along the gradients rom the portal triad to the central draining vein. Extracellular matrix (ECM) compositions, solute concentrations, oxygen availability, gene expression and metabolism vary across the liver sinusoid, and all play a role in the regulation and function of the specific cells depending on location along the gradient. The overall representation of these gradients is illustrated in Figure 1a. Oxygen and nutrient concentration decreases across Zone 1 to Zone 3. The oxygen concentration differences can promote differential hypoxia-inducible transcription factor (HIF) expression. Expression of HIFs can further modulate β-catenin and the Hedgehog (Hh) signaling pathway [28]. Recent studies have shown endothelial cells can also contribute to the regulation of these pathways for the required activation of hepatocytes in damaged livers [29,30]. Furthermore, in vivo studies have shown that variation of oxygen and nutrient gradients across the sinusoid significantly altered the endothelial structure [31,32]. 

Metabolic activity of hepatocytes as measured through the expression of cytochromes P450 (CYPs) runs opposite to both the oxygen and nutrient gradients across the zones of the lobule. These CYP enzymes have been shown to significantly change the efficacy of drugs and provide unique considerations for drug design. Some drugs are activated by CYPs in some cases increasing the hepatotoxicity of the compound. Conversely, the effects of majority of drugs are detoxified and inactive as a result of increased CYP activity [33,34]. As shown in Figure 1c, the CYP activity increases from zone 1 to zone 3. Due to this gradient across the liver lobule, the zonal pattern of hepatic toxicity differs greatly and has been previously observed in bioactivated compounds such as acetaminophen, carbon tetrachloride, bromobenzene, and chloroform [35].

The gradients across the liver zones have shown to alter the properties of LSECs. LSECs have fenestrae (window-like openings in the structure of the cell membrane) to facilitate lipoprotein traffic between the hepatocytes and the sinusoid. LSEC fenestrae are highly variable: size decreases closer to the central vein, but the number of fenestra increases. This variation has been shown to affect transvascular flux across the sinusoidal endothelium, which directly influences the transport of solutes and drugs [36].

## 3. Cells of the Liver and Resources

The liver is composed of both parenchymal cells, hepatocytes, and non-parenchymal cells, which primarily include liver sinusoid endothelial cells, Kupffer cells, hepatic stellate cells, and cholangiocytes. The surrounding structure of the vascular sinusoid is different than the capillaries of other organs. The liver sinusoid is composed of LSECs that are integrated with liver tissue resident macrophages, called Kupffer cells. The sinusoid is surrounded by a perivascular gap, known as the space of Dissé which includes hepatic stellate cells (HSCs). Surrounding the space of Dissé is the hepatic plate, which is composed of the epithelial hepatocytes.

### 3.1. Hepatocytes

Hepatocytes comprise the major population of the liver and perform many of the liver’s key functions. The hallmark of many liver diseases is direct or indirect chronic damage to hepatocytes. Depending on the selection of the appropriate hepatocyte cell line, varying phenotypic or genotypic states of different liver diseases could be investigated. In this section, we outline the properties of different hepatocytes sources for recent in vitro studies and how they have been used for different purposes.

#### 3.1.1. Non-Tumor Derived

Primary derived hepatocytes from healthy human livers provide consistent, physiological, albumin, urea, and CYP expression consistent through the culture duration [37,38,39,40]. However, limited availability of samples, sophisticated isolation procedures, restricted life span, variability, and costs limit the widespread use of primary liver cells [41]. Immortalized healthy human hepatocyte cell lines provide a more cost effective alternative to primary hepatocytes, while maintaining hepatic metabolic function and regulation representative of a healthy liver [42]. Transformed Human Liver Epithelial (THLE)-2 and THLE-3 cells are hepatocyte cell lines from healthy human donors immortalized using SV40 large T-antigen and transfected with CYP enzymes [43,44]. This provides them with a more comparable metabolic activity to native human hepatocytes, but they present with less comparable gluconeogenesis and hepatokine expression than some tumor derived hepatocyte lines [43,45].

Pluripotent stem cells (iPSCs) differentiated to hepatocytes also showed stable albumin, urea, and CYP450 activity [46,47]. However, iPSCs require specific induction factors for a long duration to obtain differentiated hepatocytes [48]. Furthermore, cultures that have a duration for longer than two weeks shows a reduction of functional protein expression in iPSC hepatocytes [49].

#### 3.1.2. Cancer-Derived Immortalized Cell Lines

Immortalized, cancer-derived hepatocytes are commonly used in microfluidic systems due to their relative availability and ease of culture. Several studies summarized the representability of commercially available HCC cell lines based on their gene expression in comparison with primary human HCC samples [50,51,52]. Bearing in mind that HCC is a highly heterogeneous group of neoplasms, HepG2 has the highest Spearman correlation to primary HCC patient samples based in this comparison making it one of the most frequently utilized. C3A, a clonal derivative of HepG2, is also commonly used and demonstrates strong contact inhibition of growth and high albumin production.

HepG2 or C3A HCC cell lines are often used as a proxy for healthy hepatocytes due to the limited availability of commercially available non-tumor derived hepatocyte cell lines. However, one of the primary concerns with the HCC cell lines is the hepatocyte metabolism. The metabolic activity of hepatocytes in a diseased liver is dysregulated, typically shown by reduced CYP expression in HCC derived cell lines [50]. This can have a significant impact in drug testing and validation as many drugs are differentially affected by a reduction in hepatocyte metabolism [33].

To maintain the ease of culturing HCC derived hepatocytes and allow study of physiologically relevant hepatocyte metabolic activity, researchers developed a subline of HCC cell lines with enhanced expression of CYP3A4 mRNA and CYP3A4-mediated activity such as C3A subline 28 (C3Asub28) [25]. C3Asub28 demonstrates a 7-fold higher CYP3A4 expression than the HepG2 cell line when cultured as a monolayer [53] allowing these sublines to more closely model the drug metabolic activity of healthy human liver hepatocytes for use in studies involving hepatocyte metabolic activity [33,34].

Other HCC derived cell lines include HuH-7 due to its permissibility for Hepatitis C (HCV) infection, allowing the study of HCC and HCV [54]. It also presents with an overexpressed tumor suppressor p53 gene, unique from other available HCC cell lines. Additionally, SNU449 and Hep3B2.1.7 cell lines derived from patients with hepatitis B (HBV) in the hepatocyte genome, have been utilized and may demonstrate potential mutations relevant to the disease [50].

### 3.2. Stellate Cells

The persinusoidal space between endothelial cells and hepatocytes (Space of Dissé) is composed of liver-specific fibroblasts called hepatic stellate cells (HSCs). Due to activation and collagen/fibrin deposition of stellate cells, the space of Dissé is the best location in the liver to investigate ECM-related diseases such as fibrosis. The process of fibrosis is primarily regulated by HSCs via their differentiation to myofibroblasts. Deposition of collagen/fibrin fibers can increase the stiffness of the liver ECM three to five fold [55,56].

HSCs in the healthy liver state are responsible for storing liposomes, retinoids, and Vitamin A. Recently, LX-1 and LX-2 cells were developed and commercialized; these cells have been used to investigate hepatic fibrosis, cirrhosis, and fatty liver diseases [57]. Other immortalized stellate cells such as hTERT-HSC, GREF-X, LI90, TWNT-1 have been used, but LX-2 cells remain the most popular stellate cell line for hepatic fibrosis studies since this cell line has been characterized and sustained key features of cytokine signaling, neuronal gene expression, retinoid metabolism, and fibrogenesis [58]. Alternatively, freshly isolated primary HSCs have been utilized, however, self-activation during the culture period and non-matching gene expression levels have been problematic in recent studies [57].

### 3.3. Kupffer Cells

Kupffer cells (KC) are the tissue resident liver macrophages responsible for the release of many effector molecules in response to liver disease or exposure to exogenous substances. They serve as the first line of defense for pathogens entering the liver and preventing initial viral replication [59]. However, in the case of the diseased liver, KCs play a significant role as mediators of liver injury and repair. They function paradoxically as both protectors against and initiators of many of the harmful effects of chronic liver disease. KCs are the source of the majority of pro-inflammatory molecules in the liver which can further hepatic damage [60,61,62]. However, they can also behave in an immunosuppressive manner in liver cancers, enhancing cancer metastasis, angiogenesis, and chemotherapy resistance [63,64]. Currently, there are no immortalized Kupffer cells from patient samples; they have only been isolated from mice and rats. Cryopreserved Human Kupffer Cells (HUKCCS) cell line is the only available primary KC isolated from a human, but it has not been immortalized, making its use cost-prohibitive. Because of these limitations, the THP-1 human monocytic leukemia cell line has been used as a proxy for tissue resident Kupffer macrophages. Studies show that differentiation of this cell line with phorbol-12-myristate-13-acetate (PMA) can portray Kupffer-like cytokine expressions [65]. Less commonly, U-937, a pro-monocytic, human myeloid leukemia cell differentiated into macrophages using 1,25-dihydroxyvitamin D3 (VD_3_), has also been utilized as a Kupffer cell analog for in vitro study.

### 3.4. Endothelial Cells

LSECs regulate the exposure of hepatocytes to sinusoidal blood. This cell type usually demonstrates fenestrations and endocytosis abilities. LSECs not only function as the permeable barrier between the circulating blood, and resident liver cells, they have been shown to help maintain the native morphology of hepatocytes in vitro and help upregulate functional protein secretion such as albumin, urea, and numerous drug metabolism enzymes [10,11]. Conditions in a diseased liver have been shown to significantly alter LSEC integrity and morphology, and these subsequent LSEC alterations play an important role in disease progression. Damaged LSECs can lose fenestration and release proinflammatory cytokines, such as (Transforming growth factor beta)TGF-β, which can stimulate hepatocytes and stellate cells to further the damage to the liver, and participate in disease-mediated angiogenesis [31,66]. In addition, during hepatic injury LSECs serve as one of the regulators of hepatic fibrosis progression, a main driver of liver regeneration, and contribute to waste management [12]. As a result, it is vital to investigate the exact role that the LSECs plays both in response to, and during the progression of, chronic liver disease. Although LSECs have been isolated from patients for primary culture, these cells have been reported to be unstable, difficult to cryopreserve, and demonstrate a lack of vascular endothelial cadherin (VE-cadherin) expression when cultured together with the other major hepatic cell lines [67,68]. Alternatively, several groups used human umbilical vein endothelial cells (HUVECs) but this cell line lacks of receptors such as CXCR3 and EGFR, leading to variable results [69,70]. Another primary endothelium that has been used is human microvasculature endothelial cells (HMVECs), this cell line has improved VE-cadherin expression compared to other primary endothelial cell lines. However, lumen diameter of vascularized in vitro systems is fairly large (≅ 100 μm) compared to a typical microvasculature, which makes the use of HMVECs less feasible than HUVECs or LSECs.

**Figure 2:**
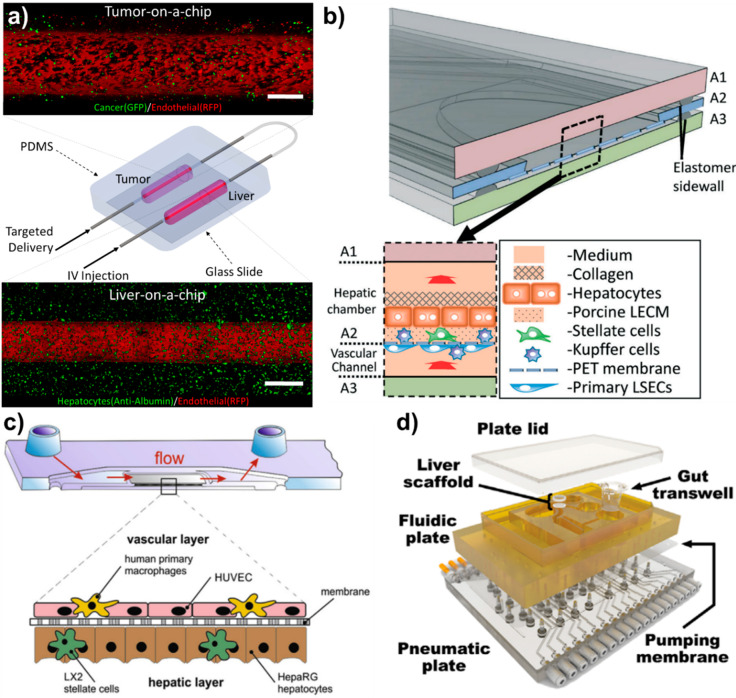
Novel vascularized liver-on-a-chips. (**a**) Vascularized liver-on-a-chip incorporated with tumor-on-a-chip to investigate enhanced permeability retention (EPR) effect of different nanoparticles under physiological wall shear stress. Flow direction could be controlled to simulate targeted delivery (tumor to liver) and IV injection (liver to tumor). Scales are 500 μm. Reproduced from [8] with permission from Wiley. (**b**) The vascularized liver acinus microphysiology system (vLAMPS). The vLAMPS is constructed from 3 glass layers. The intermediate layer contains an elliptical opening with a Polyethylene terephthalate (PET) membrane with pores that spans the opening and is attached to the bottom. Reproduced from [67] with permission from the Royal Society of Chemistry. (**c**) The three-dimensional liver organoid consists of a vascular layer formed by endothelial cells and primary macrophages, and a hepatic layer comprising hepatocyte-like HepaRG cells co-cultured with stellate cells. The space of Dissé (SD) is mimicked by the biochip-embedded membrane serving as a scaffold allowing cell-cell communication through its pores. Reproduced from [100] with permission from Elsevier. (**d**) Exploded view of a multi-MPS platform. The top plate (shown in yellow polysulfone) contains MPS compartments and distributes culture medium through micromachined channels and pumps on its bottom face. The bottom plate (shown in clear acrylic) distributes compressed air and vacuum to small ports below each pump/valve chamber. Reproduced from [94] with permission from Wiley.

Immortalized cells provide several advantages over primary cells: they feature an extended lifespan, are functionally stable, and are a cost-effective alternative to primary cells. Immortalized liver sinusoid endothelial cells, TMNK-1, have been co-cultured with hepatocytes to investigate regulation of growth factors and inflammatory expressions [71], cytokine expression roles on liver regeneration [72], migration in response to angiogenic factors, formation of vascular tubes [73,74], and upregulation of epidermal growth factor (EGFR) under interaction with different cancer phenotypes [75]. Alternatively, human foreskin endothelial cells (HMEC-1) show improved hepatocyte albumin secretion when co-cultured with hepatocytes [76]. Telomerase-immortalized human microvascular endothelial cells (TIME) have also been co-cultured with both healthy and cancerous hepatic cell lines to reproduce a delineated enhanced permeability retention (EPR) effect as well as to quantify the influence of cirrhosis and inflammation in vascular health [8,9] (Figure 2a).

### 3.5. Other Nonparenchymal Cells

In addition to hepatocytes, stellate, Kupffer, and endothelial cells, bile duct cells and other immune cells such as T-cells, play roles in different disease states in liver. Bile duct cells (cholangiocytes) are the epithelial cells surrounding the bile duct and are responsible for the modification of hepatocyte-derived bile processes regulated by hormones, peptides, nucleotides, neurotransmitters, and other molecules along with intracellular signaling pathways and cascades [77]. Currently, two immortalized cholangiocyte cell lines (H69 and NHC) have been used in in vitro studies [78]. Both primary human and rat cholangiocytes have been used in various studies [22]. Additionally, T-cells have been incorporated for investigation of potential immunotherapy applications. For example, TCR T-cells were used to investigate immunosuppressive potential toward HBV targeting [79].

## 4. Mechanical Properties of the Liver

Mechanical properties of the extracellular matrix regulate cellular functions, such as functional protein expression, proliferation, mobility, and chemoresistance [80]. The human liver is a relatively soft organ typically presenting with a stiffness in the range of 400-600 Pa with specific compression modulus under 5%, 10%, and 15% preload strains of 640 ± 80 Pa, 1080 ± 160 Pa and 2000 ± 630 Pa, respectively [81]. Fibrotic livers, in general, feature higher stiffness levels than healthy livers from increased collagen deposition in the ECM. Yeh et al. found in their comprehensive study on a maximum fibrotic stiffness of 1650 ± 110 Pa, 4930 ± 930 Pa and 19980 ± 6950 Pa under 5%, 10%, and 15% preload strains, respectively [81,82]. HCC, the most common form of liver cancer, is commonly associated with chronic liver disease ending in fibrosis and cirrhosis. HCC microenvironment stiffness is reported to be 3000 Pa and 12100 Pa for 5% and 10% preload strains, respectively. Considering the potential factors for HCC, such as hepatitis C, hepatitis B, cirrhosis, HIV and alcoholic liver disease, liver stiffness has been reported in the range of 10–25 kPa [83,84,85,86,87].

While bulk stiffness measurements of the liver through compression or rheological testing has provided insight to the overall mechanical properties of the liver, recent studies utilizing atomic force microscopy (AFM) have provided more precise measurements of how the stiffness of individual fibers varies spatially across the liver lobules, zones, and fibrotic regions. However, it must be noted that AFM measurements will provide fiber stiffness, which may be different than the compression modulus measurements. Through AFM, it has been shown that stiffness in fibrotic regions are significantly higher (1–6 kPa) compared to healthy regions of the liver (150 Pa) [88]. Alternatively, magnetic resonance elastography (MRE) and vibration-controlled transient elastography (VCTE) were used to measure healthy liver stiffness [89]. In these measurements, normal liver stiffness has been reported as 2 kPa and 2–6 kPa for MRE and VCFE methods, respectively. Another AFM-based method to determine liver matrix stiffness showed fibronectin-null livers upregulated local TGF-β and lysyl oxidase activity, which induced myofibroblasts to accumulate disorganized and dense ECM networks [90]. This activation increased elastic modulus by 55% in fibronectin-null livers compared to control (5.13 ± 0.55 kPa in mutant versus 3.31 ± 0.84 kPa in control) [89,91].

## 5. Vascularized Liver-on-a-Chip Disease Models

Commonly used in vitro models to study chronic liver disease utilize 2D hepatocyte monolayers [73], isolated liver bioreactors [92], liver spheroids, and microfluidic devices [13]. Liver microfluidic devices, or liver models that incorporate flow, are typically considered liver-on-a-chip devices. However, due to the limited research of the hepatic vasculature, many of the studies presented have yet to include microfluidics. Because of the low cost and ease of handling, 2D culture has been widely used to study the liver, but co-culture with endothelial cells cannot be incorporated in a complex, and native architecture with the presence of flow or ECM due to the lack of lumen and accessibility of flow influx and outlets. Bioreactors can represent the cellular complexity but non-tunability of the system limits the use to investigate different states of liver diseases and endothelium dysfunction. Many recent studies show that endothelial cells can be incorporated to spheroids and liver-on-a-chips with the presence of representative ECM to investigate endothelium functionality by co-culturing with hepatocytes alone or in addition of other NPCs [7,8,9,13]. Formation of functional endothelium can be accomplished in several days with or without the influence of flow. However, the incorporation of flow in microfluidic devices with liver endothelial cells has proved to be a vital addition to maintain the phenotype and function of liver specific cells. Lee et al. 3D printed endothelial cells, HUVEC, and hepatocytes, HepG2, co-cultured on a polycaprolactone (PCL) surface [7]. In this study, the addition of HUVECs co-cultured with HepG2 improved albumin and urea secretion over HepG2 alone. The contribution of flow in the same system improved both albumin and urea release. Additionally, they found that the presence of shear stress induced by flow improved live cell population of hepatocytes and endothelial cells compared to 2D static culture. Our previous studies have previously demonstrated that incorporating flow and increasing wall shear stress in a cylindrical vessel can help to form tight endothelial junctions and confluent endothelium lumen (Figure 2a) [8,9,93]. Creating a functional endothelium is vital, as the influence of tight junctions and confluent endothelium regulates the permeability of solutes and nutrients to hepatocytes. In the following sections, we will describe how vascularized liver-on-a-chips have been used to investigate the role of the hepatic vasculature in chronic liver diseases such as inflammation, fatty liver, viral hepatitis, fibrosis/cirrhosis, and HCC. Major findings in presented liver-on-a-chip models were summarized in Table 1.

### 5.1. Inflammation

Hepatic inflammation is the leading factor of tissue damage in the liver and is commonly associated with chronic liver diseases such as viral hepatitis, the progression of non-alcoholic fatty liver disease (NAFLD) to non-alcoholic steatohepatitis (NASH), and HCC. Inflammation in hepatocytes is commonly induced with exogenous lipopolysaccharide (LPS) administration either at low (1–2 ng/ml) or high (2–10 ng/ml) levels to produce mild to severe inflammation in in vitro liver models [60,94,95]. LPS treatment has shown to alter immune and defense responses, cytokine-mediated, and interferon signaling pathways in liver-on-a-chip (Figure 2d) [94]. However, inflammatory cytokines such as tumor necrosis factor alpha (TNF-α), IL-1β, IFN-γ, and IL-6 have also been used to stimulate inflammation in liver [96,97]. On the other hand, liver inflammation in an in vitro environment can be approximated by increasing the number of Kupffer cells in the microenvironment by mimicking the natural recruitment seen in liver inflammation. Studies have shown that when the liver is inflamed, hepatocyte to Kupffer ratio increases from 10:1 to 10:4, which subsequently increases the proinflammatory cytokine expression [98,99].

**Table 1:** Major findings reported in vascularized liver-on-a-chips.

Disease Model	Cell Lines	Major Findings	Reference
Inflammation Fibrosis/Cirrhosis HCC	HepG2 C3Asub28 LX-2 THP-1 TIME	● Cirrhosis and inflammation increased vascular damage, and permeability due to upregulated inflammatory cytokines. ● High CYP3A4 expressing HCC cells increased vascular damage and permeability.	Özkan et al. [9]
Inflammation	Primary hepatocytes LX-2 THP-1 LSECs	● LSECs activated by LPS promoted α-SMA expression of stellate cells. ● α-SMA expression of activated stellate cells was higher in zone 3 compared to zone 1.	Li et al. [67]
Inflammation Fibrosis/Cirrhosis	Rat PMVECs HPMECs HCMECs HDMECs	● Increase in matrix stiffness upregulated production of podosomes, actin-based structures involved in cell adhesion, migration, invasion, and ECM degradation, independent of classical podosome inductors VEGF and TGF-β.	Juin et al. [101]
Inflammation Fibrosis/Cirrhosis	LSECs	● Increase in substrate stiffness upregulated the expression levels of VCAM-1 and ICAM-1 in LSECs.	Natarajan et al. [102]
ALD/NAFLD	HepG2 LX-2 EAhy926 U937	● Increase of ethanol concentration decreased VE-cadherin expression of endothelial markers, resulting in a potentially leakier vasculature. ● eNOS was downregulated when exposed to high doses of exposure to ethanol.	Deng et al. [103]
ALD/NAFLD	Rat hepatocytes HUVECs	● Co-culture of hepatocytes with HUVECs doubled chain fatty acid family members compared to a monoculture.	Takayama et al. [104]
Hepatitis B	Rat Hepatocytes BAECs	● Co-culture with endothelial cells sustained longer and consistent secretion of urea and also improved and sustained liver specific differentiation markers such as albumin, transferrin hepatocyte nuclear factor 4, and β-actin compared to hepatocytes alone.	Kang et al. [105]
Hepatitis B	Rat Hepatocytes BAECs	● HBV infected hepatocytes and BAECs showed that hepatocytes with HBV lost their native morphology within a week without the presence of BAECs.	Kang et al. [106]
Fibrosis/Cirrhosis	LSECRat KCs	● HBV passing through endothelium fenestrate and infecting the hepatocytes. ● Increase in matrix stiffness decreased LSEC proliferation and increased the size of adhesion sites, loss of fenestrae, expression of CD31, while substantially altering cell morphology.	Ford et al. [107]
Healthy Liver HCC	MDA-MB-231 THLE-3 C3Asub28 TIME	● Endothelial co-culture with HCC cells experience EPR effect unlike co-culture with healthy liver. ●70 kDa dextran (size of chemotherapy+NP) accumulated less in healthy liver than 3 kDa dextran particles (plain chemotherapy).	Özkan et al. [8]

Inflammation has been found to be a significant influence in LSEC function and has been investigated in several vascularized liver-on-chip platforms. Clark et al. found that inflammation induced by LPS upregulated gene expression, such as IL-7, MIP-1a, VEGF-A, and TECK of endothelial cells, which was cultured on polyethylene glycol diacrylate 3D hydrogel scaffolds, mediated pro-inflammatory responses [108]. Upregulation of these pro-inflammatory mediators is reported as the clinically relevant hepatic endothelium injury, which is associated with non-functional endothelium barrier functionality. Li et al. constructed a glass-based vascularized liver-on-a-chip to investigate how LSECs activated by LPS alter the stellate cell activation in the microenvironment, and consequently how collagens are deposited in different zones of liver [67] (Figure 2b). This study showed that activation of LSECs caused higher expression of α-SMA-expressing stellate cells in a vascularized microfluidic platform. In addition, α-SMA expression of stellate cells was higher in zone 3 compared to zone 1, which resulted in a high number of fenestrations on the LSECs. The lack of tight junctions and an increase in fenestrations in activated LSECs has been observed to cause polymorphonuclear leukocyte (PMN) migration from the vasculature to the vicinity of hepatocytes. Gröger et al. demonstrated that inflammation could be stimulated using toll-like receptor (TLR) in microfluidically-supported vascularized organoid liver model, where hepatic and vascular channels were separated with polyethylene terephthalate [109]. This study showed TLR-stimulation induced the release of pro- and anti-inflammatory cytokines and decreased expression of VE-cadherin and ZO-1 in endothelial cells and disruption of the endothelial barrier. The study also showed LPS-treatment induced monocyte adhesion/migration through leaky endothelial cells to the hepatic region. This release of endothelial SICAM-1 and sVCAM-1, triggered an increase in IL-1β, IL-6, and TNF-α levels of the monocytes of the system and further increased the vascular interruption. An alternative to LPS, the C-terminal fragment of alpha-1-antitrypsin (CAAP48) has been introduced and used to initiate inflammation and investigate multi-cellular inflammatory response in vascularized liver-on-a-chip systems under the influence of physiological flow conditions [110]. The molecular mechanism of CAAP48 is very similar to other stimulants, such as upregulation of IL-6, IL-10, TNF-α, and IL-1β cytokines. In this study, CAAP48 was shown to decrease VE-cadherin and F-actin expression compared to control by 35% and 25%, respectively. Rennet et al. created a cyclic olefin copolymers (COC) system with microfluidic flow conditions and demonstrated that sepsis (severe inflammation) caused hepatocellular dysfunction and promoted the release of both pro- and anti-inflammatory cytokines [100] (Figure 2c). This followed the interruption of endothelial viability and decreased the expression of the hepatic transporter (MRP2). Overall, the induction of an inflammatory stimulant in vascularized liver-on-a-chips increased inflammatory cytokines expressed by all cells in the microenvironment, including endothelial cells. The expression of inflammatory cytokines leads to a decrease of endothelial gene expression related to cell-cell junction integrity. However, endothelium leakiness under the influence of inflammation has yet to be thoroughly reported quantitatively (such as vascular permeability, vascular porosity and size of fenestrae).

### 5.2. Fatty Liver Diseases and Alcoholic Liver Disease

Due to the prevalence of obesity and Western diets, non-alcoholic fatty liver disease (NAFLD) has emerged as a common chronic liver disease. It is expected to continue to increase as the incidence of obesity rises [111]. NAFLD resembles alcoholic liver disease (ALD) due to the similar pathological presentation of the accumulation of lipids within hepatic cells. This lipid accumulation correlates to reduced perfusion and changes in the underlying matrix structure that may impact the transport and efficacy of therapeutics [112,113]. In general, alcohol injury caused by ALD has been generated in in vitro systems by circulating low dose ethanol through the microchannels. To induce NASH or NAFLD, different forms of free fatty acids (FFAs) observed in Western diets have been used including palmitic acid, linoleic acid, and oleic acid [114,115,116].

The effect of ALD and NASH/NAFLD on the liver endothelium has been investigated in several studies. Deng et al. investigated the effect of ALD on EA.hy926 endothelial cells [103] by perfusing ethanol at different concentrations through vascularized liver-on-a-chip. A gradual increase of ethanol concentration decreased VE-cadherin expression of endothelial markers, demonstrating that alcohol damages the tight junctions resulting in a potentially leakier vasculature. Moreover, the same study showed that endothelial nitric oxide synthase (eNOS), which inhibits activation of stellate cells, was downregulated when exposed to high doses of exposure to ethanol. Also, the activation and proliferation of stellate cells due to ethanol exposure was demonstrated in the same device by quantifying upregulation of alpha-smooth muscle actin (α-SMA) expression. Another study by Lasli et al. incorporated HUVECs in a vascularized liver spheroid model to investigate lipid accumulation and progression of NAFLD [117]. This study showed that incorporation on HUVECs in the spheroid model increased the accumulation of lipids in the spheroid core compared to a system without HUVECs. Another study by Takayama et al. co-cultured HUVECs with rat hepatoma Fao cells to investigate the regulation of fatty acids compared to monoculture [104]. As a result of this study, it has been found that a co-culture with HUVECs doubled chain fatty acid (ELOVL) family members compared to a monoculture. Overall, these findings show endothelial cells are significantly affected by the compounds leading to ALD or NAFLD/NASH and alter the lipid accumulation of hepatocytes and their further risk for steatosis and a potential irreversible liver disease. 

### 5.3. Viral Hepatitis

Viral Hepatitis, primarily hepatitis B (HBV) and hepatitis C (HCV) have long remained one of the more prevalent types of chronic liver disease. While HCV is more common in the United States, Europe and Japan, HBV accounts for roughly 50% of the progression to HCC worldwide [118,119]. In these cases we see not only the direct introduction of viral DNA (such as in the case of HBV) or indirect regulation of cell function (such as in the case of HCV), but an increased rate of cell turnover, which can result in compounding genetic mutations [120,121]. Moreover, HBC and HCV have been associated with HCC since approximately 75% of the HCC cases are due to chronic hepatitis infection. HBV in vitro studies are commonly performed by infecting primary or immortalized hepatocytes with patient-derived HBV [122]. Several existing hepatocyte cell lines are derived from patients with prior active HBV infection with HBV DNA incorporated into the host genome [51]. Unfortunately, there is currently no existing vascular model that closely investigates the host modulation due to HCV. Great difficulty has been encountered with in in vitro propagation of HCV, and current research relies on the development of sub-genomic replicons that recapitulate intracellular replication, but permit easy culture, to quantify the viral replication, host antiviral response, and drug discovery [123]. Current research in HCV replication utilizes the HuH-7 cell line, the most permissible cell line for HCV replicon culture, to quantify the role the host plays in viral replication [124]. Studies have not yet fully elucidated the impact the virus has on host function. For that reason, there have been no investigations incorporating HCV in liver-on-a-chips. 

The very first HBV viral replication study was carried out by Sodunke et al., where a system composed of only hepatocytes were transfected with an HBV-genome cDNA or infected with the viral genome expressed from a recombinant adenovirus [125]. In a different system, Ortega-Prieto et al. carried out a more comprehensive HBV infection and replication study in liver-on-chip platform, which additionally incorporated Kupffer cells in their system [122]. This system recapitulated all life cycles of HBV, such as replication and maintenance, as observed in HBV patients. However, the system did not have endothelial cells to investigate the vascular component. The influence of hepatitis to the vascular component has been observed only in a few studies [105,106]. Vascularized liver-on-a-chip system composed of hepatic and vascular microfluidic channels created by Kang et al. co-cultured primary rat adrenal medullary endothelial cells (RAMECs) with hepatocytes that were subsequently infected with a recombinant adenovirus with the HBV genome, to permit infection of rat hepatocytes [105]. They demonstrated that the hepatocytes retained their phenotype through the study and that this platform could be utilized to study the replication of HBV in a physiological multi-cellular environment. This study also showed that co-culturing bovine aortic endothelial cells (BAECs) with primary rat hepatocytes and HBV sustained longer and consistent secretion of urea and also improved and sustained liver specific differentiation markers such as albumin, transferrin hepatocyte nuclear factor 4, and β-actin in co-cultured hepatocytes for 21 days compared to hepatocytes alone. In a further study with HBV infected hepatocytes and BAECs, the group showed that hepatocytes with HBV lost their native morphology within a week without the presence of BAECs. Additionally, they carried out the HBV infection in the chip, and reported HBV passing through endothelium fenestrate and infecting the hepatocytes [106].

### 5.4. Fibrosis and Cirrhosis

Progressive liver fibrosis with the development of cirrhosis is a feature in the later stages of most chronic liver diseases. In response to chronic liver injury, the common progression of a healthy liver to fibrosis and eventually cirrhosis is a multifaceted process that results in a complex histological mix of nodules and fibrous bands [126]. Chronic injury to hepatic tissue results in hepatocyte death and subsequent inflammation in the tissue from activated Kupffer and endothelial cells. The cytokines and chemokines released by these cells activates the neighboring hepatic stellate cells, promoting their differentiation into a myofibroblastic phenotype. These activated HSCs are the primary contributors to fibrosis/cirrhosis as they dramatically increase the production of α-SMA and inflammatory molecules as well as the deposition of ECM proteins directly initiating the most notable hallmark of fibrosis/cirrhosis, the increased stiffness of the tissue microenvironment [81,82]. Although this increase in microenvironmental stiffness has been classically seen as a consequence of chronic liver disease, recent research demonstrates that it is a critical factor involved in disease progression. Furthermore, the complex interplay between activated HSC’s and surrounding cells, including LSECs, enables the progression of fibrosis through the underlying inflammation and tissue stiffness modulated cellular mechanisms.

As hepatic fibrosis is primarily regulated by HSCs, many microfluidic and 3D studies focusing on the initiation and progression of fibrosis involve the co-cultures of hepatocytes and HSCs. Leite et al., for example, utilized human hepatic organoids to study the activation and ECM deposition of HSCs in response to cellular damage of hepatocytes [127]. However, few studies have isolated the impact that fibrotic stiffening of the ECM has on cellular function directly, and even fewer have investigated this impact on liver endothelial cells [128].

The involvement of LSECs in hepatic fibrosis is complex. The fibrotic process is preceded by changes in the liver endothelial cells into a pro-inflammatory, capillarized phenotype. This change leads to further dysregulation of endothelial cells in response to advancing fibrosis. In conjunction with the underlying disease, because the liver function and structure are both considerably altered, this endothelial dysregulation results in compromised drug transport and efficacy within the liver [121]. Studies show that LSECs demonstrate phenotypical regulation in response to the substrate they are cultured on [129], and the increase in substrate stiffness, namely collagen I, can simulate LSEC-dependent angiogenesis, furthering the condensation of collagen fibers in the ECM and activation of HSCs [128].

Juin et al. and Natarajan et al. demonstrated that changes in liver endothelial phenotype can be solely driven by stiffening of the ECM without the influence of other cell types or contribution of cytokines [101,102]. Both of these investigations, however, utilized non-native ECM and neglected the impact of flow, limiting the applicability of the results to in vivo observations; Juin et al. used polyacrylamide and Natarajan et al. used polydimethylsiloxane with a collagen I coating, These studies demonstrated significant impact of matrix stiffness on LSEC adhesion molecules and properties. Juin et al. showed that matrix stiffness enhanced the production of podosomes, actin-based structures involved in cell adhesion, migration, invasion, and ECM degradation, independent of classical podosome inductors VEGF and TGF-β [101]. In addition, Natarajan et al. demonstrated that the increase in substrate stiffness correlated to a significant increase in expression levels of vascular cell adhesion molecule (VCAM-1) and intercellular cell adhesion molecule (ICAM-1) in LSECs. They also investigated the reduction of endocytic capacity of LSECs in response to being cultured on a stiffer substrate. This reveals a potential mechanism for the increase in serum levels of hyaluronic acid from fibrosis resulting from chronic liver damage [102]. Ford et al. utilized this principle and expanded the investigation of the influence of matrix stiffness on LSEC behavior to tissue native ECM. They utilized collagen I hydrogels with differing Young’s moduli to mimic liver stiffness at 6 kPa (healthy liver) and 36 kPa (fibrotic liver) [107]. They found that an increase in matrix stiffness decreased LSEC proliferation and increased the size of adhesion sites, loss of fenestrae, expression of CD31, while substantially altering cell morphology.

### 5.5. Hepatocellular Carcinoma

Hepatocellular carcinoma (HCC) is the most common form of liver cancer and the second leading cause of cancer related deaths. Recent findings show that increase in arterialization, and sinusoid capillarization, hypervascularity, and angiogenesis makes HCC detrimental to liver’s vascular health [130]. Literature on in vitro HCC models with endothelial cells is limited. Chiew et al. showed that HepG2 cells led HUVEC-C3 cells to differentiate and form tubular structures and protrusions [131]. Chiew et al. further found that HUVEC-C3 differentiation could not be achieved with the conditioned media, in contrast with a co-culture with HepG2 cells. It has also been shown that endothelial cells were more resistant to anti-angiogenic inhibitors, such as SU5416, sorafenib, U0126, SP600125, LY294002, SB202190 and Y27632, under co-culture conditions compared to monoculture. Özkan et al. found that C3Asub28 cells increased endothelial damage by 3.62-fold (p < 0.001) compared to healthy liver-on-a-chip [8]. The demonstration of endothelial damage due to the EPR effect resulted in an increase in vascular permeability.. The increase of endothelial leakiness has been related to transendothelial migration due to the signals expressed by cancer cells and cancer cell invasion [132,133].

## 6. Future Directions

In addition to commonly studied liver diseases, several other chronic liver diseases have a great impact on global health. In this section, we will describe these diseases and identify areas for future investigation. Cholangiocarcinoma (CCA) is a rare type of cancer that originates from the epithelial cells of the biliary ducts in the liver and significantly alters the transport of bile drainage. It exists on the spectrum of neoplasia with HC and occasionally coexists with HCC. Although CCA is rare, it presents a grim prognosis as the 5-year survival rate is roughly 25% [134]. Given the high and increasing mortality rate, microfluidic organ-on-a-chip technology can be used to recapitulate important physiological function to investigate cell-cell and cell-ECM functions. To address this problem, Yu et al. created the first vascularized bile duct-on-a-chip to investigate barrier functions and alternations under the influence of Glycocalyx treatment [22]. They found that the bile duct-on-a-chip’s basolateral side was more sensitive than the apical side to the treatment that shows a protective adaptation of the apical surface that is normally exposed to bile.

Another vascular complication of disease that has not been investigated in detail is hepatic angiogenesis in HCC. The hypervascularity and other vascular abnormalities, such as arterialization and sinusoidal capillarization, of HCC tumors results in angiogenesis and the further growth of tumor mass [135]. Current approaches and clinical trials rely on novel strategies and drugs, such as sorafenib, to inhibit Raf-1, B-Raf and VEGFR-2 multikinases to obstruct vascular growth [136]. Numerous vascularized organ-on-a-chips have investigated the angiogenic activity of endothelial cells in response to breast [137] and pancreatic cancer [138] as well as artificial stimulants [139,140]. Several 3D in vitro Matrigel models were introduced to investigate angiogenic cytokine inhibition correlation with endothelial response [131,141], but the microfluidic technology, control of vascular component and contribution of wall shear stress were not incorporated in those systems.

Lastly, drug-induced vascular damage and dysfunction causes dysregulation of homeostatic functions in the liver by increasing nitric oxide and reactive oxygen species activity [142]. Exogenous compound-induced liver injury in animals tests is the a common reason drug trials fail, which has cost nearly $2 billion annually in the pharmaceutical industry [143]. Due to drugs accumulating in liver, hepatic injury has been seen in many cases. Non-selective damage on endothelial cells, however, has not been well-investigated [33,144]. In this limited field, Özkan et al. used a novel vascularized HCC-on-a-chip system to test different doxorubicin delivery methods; in particular, they investigated intravenous (IV) injection and transarterial chemoembolization (TACE) to determine treatment efficacy and non-selective damage on endothelial cells [9]. This study showed that delivering drugs through the TACE method is more beneficial than IV to sustain blood vessel endothelium integrity.

Studies of the hepatic vasculature in the context of flow are still limited. Many microfluidic liver-on-a-chip models either neglect the significant role of the hepatic vasculature, or do not investigate the role of chronic liver diseases at all. Even though the very first microfluidic liver-on-a-chip was introduced thirteen years ago [145], new liver-on-a-chips continue to surface with very few carrying out extensive mechanistic studies. There remains a significant gap in the research and these models highlight the importance of the vasculature in response to, and in the progression of chronic liver diseases. While some may be limited in scope, they provide a clear path for expansion and other microfluidic liver-on-a-chips to incorporate these principals in future studies. By expanding this research into fully realized vascularized microfluidic platforms, we will be able to understand the vascular nuances that prove difficult to investigate in vivo. This will significantly contribute to the mechanistic knowledge of the driving factors of chronic liver disease. Furthermore, the use of these models for drug discovery in a more physiological in vitro setting will provide a more instructive research tool to test and develop novel, targeted therapies for chronic liver diseases.
